# Slit/Robo Signaling Regulates Multiple Stages of the Development of the *Drosophila* Motion Detection System

**DOI:** 10.3389/fcell.2021.612645

**Published:** 2021-04-21

**Authors:** Pablo Guzmán-Palma, Esteban G. Contreras, Natalia Mora, Macarena Smith, M. Constanza González-Ramírez, Jorge M. Campusano, Jimena Sierralta, Bassem A. Hassan, Carlos Oliva

**Affiliations:** ^1^Department of Cellular and Molecular Biology, Faculty of Biological Sciences, Pontificia Universidad Católica de Chile, Santiago, Chile; ^2^Department of Neuroscience and Biomedical Neuroscience Institute, Faculty of Medicine, Universidad de Chile, Santiago, Chile; ^3^Institut du Cerveau-Paris Brain Institute (ICM), Inserm, CNRS, Hôpital Pitié-Salpêtrière, Sorbonne Université, Paris, France

**Keywords:** nervous system development, cell migration, axon guidance, Slit-Robo pathway, *Drosophila melanogaster*

## Abstract

Neurogenesis is achieved through a sequence of steps that include specification and differentiation of progenitors into mature neurons. Frequently, precursors migrate to distinct positions before terminal differentiation. The Slit-Robo pathway, formed by the secreted ligand Slit and its membrane bound receptor Robo, was first discovered as a regulator of axonal growth. However, today, it is accepted that this pathway can regulate different cellular processes even outside the nervous system. Since most of the studies performed in the nervous system have been focused on axonal and dendritic growth, it is less clear how versatile is this signaling pathway in the developing nervous system. Here we describe the participation of the Slit-Robo pathway in the development of motion sensitive neurons of the *Drosophila visual* system. We show that Slit and Robo receptors are expressed in different stages during the neurogenesis of motion sensitive neurons. Furthermore, we find that Slit and Robo regulate multiple aspects of their development including neuronal precursor migration, cell segregation between neural stem cells and daughter cells and formation of their connectivity pattern. Specifically, loss of function of *slit* or *robo* receptors in differentiated motion sensitive neurons impairs dendritic targeting, while knocking down *robo* receptors in migratory progenitors or neural stem cells leads to structural defects in the adult optic lobe neuropil, caused by migration and cell segregation defects during larval development. Thus, our work reveals the co-option of the Slit-Robo signaling pathway in distinct developmental stages of a neural lineage.

## Introduction

The development of every tissue is orchestrated in a sequence of discrete steps. Strikingly, a small repertoire of signaling pathways, which are co-opted throughout development, regulate the formation of tissues and organs. An example of this takes place in the developing nervous system, in which a neuroepithelium gives rise to differentiated neurons (Gotz and Huttner, 2005; Holguera and Desplan, 2018; Tiberi et al., 2012). Several cellular mechanisms participate in generating the great diversity of neurons that populate both central and peripheral nervous systems (Brand and Livesey, 2011; Holguera and Desplan, 2018). Neural stem cells progress through multiple stages including different modes of division, such as amplification and self-renewal. Furthermore, during the development of the vertebrate brain many neural precursors and neurons migrate to reach specific regions in the nervous system (Buchsbaum and Cappello, 2019). How a limited number of signaling pathways account for the complex transition in cellular processes involved in neurogenesis is not completely understood.

**FIGURE 1:**
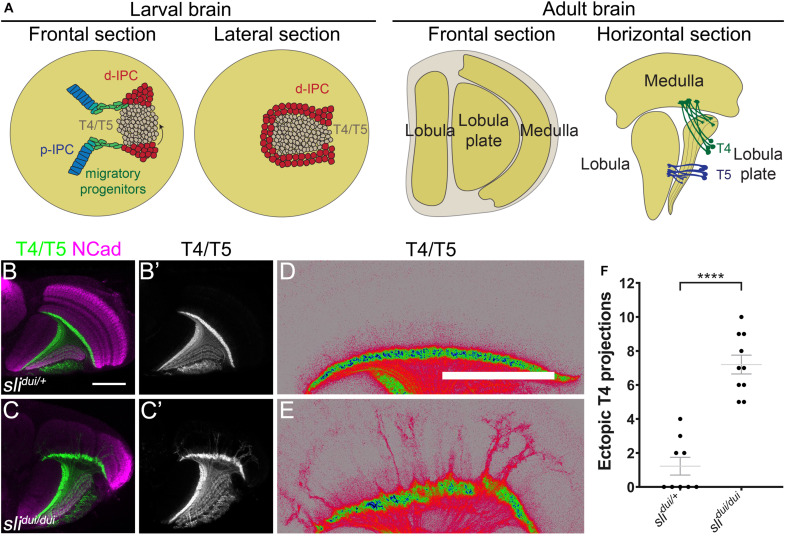
*Slit* is required for T4 dendrite targeting. **(A)** Schemes illustrate the different views of larval and adult optic lobes, showing the cell populations of interest. d-IPC, distal inner proliferation center; p-IPC, proximal inner proliferation center; T4, T4 neurons; T5, T5 neurons. **(B–E)** Horizontal sections of panels **(B–D)** *sli*^dui/+^ heterozygous (Control) and **(C–E)** *sli*^dui/dui^ adult optic lobes stained against NCad (magenta) and expressing CD4-tdTomato under the control of *bab2-GAL4* driver to label T4 and T5 neuronal projections (green, gray and pseudocolor). **(D,E)** are high magnification views of panels **(B,C)** with pseudocolored T4/T5 neurites. **(F)** Graph showing the number of T4 mistargeting projections in *sli*^dui/+^ and *sli*^dui/dui^ adult optic lobes. Student’s *t*-test was performed. **** is *p*-value = 5.04 × 10^–7^. Scale bars represent 50 μm.

The visual system is a powerful model for understanding different aspects of neurogenesis. In insects, such as *Drosophila melanogaster*, the visual system is comprised by a compound eye (bearing the retina) and the optic lobe. The *Drosophila* retina contains around 800 repetitive units, called ommatidia, each formed by eight photoreceptors (R-cells) and 20 accessory cells. The optic lobe contains four ganglia: the lamina, medulla, lobula and lobula plate. The optic lobe develops from the optic placode generated during early embryonic development. In larval stages, two neurogenic regions differentiate and separate from each other into the outer proliferating center (OPC) and the inner proliferating center (IPC). While the OPC gives rise to the lamina and the outer medulla, the IPC generates the inner medulla, lobula and lobula plate (Apitz and Salecker, 2014; Contreras et al., 2019; Courgeon and Desplan, 2019; Hofbauer and Campos-Ortega, 1990; Sato et al., 2019). The lobula plate is a critical component of the *Drosophila* motion detecting system, with different neuronal populations that respond to either dark or bright edges (Maisak et al., 2013). During larval development, the IPC splits into three main regions. The closest to the central brain, includes two epithelial subdomains: proximal (p-IPC) and superficial (s-IPC). From the p-IPC, streams of migrating progenitors connect with the distal (d-IPC) on the opposite side of the optic lobe (see Figure 1A). The presence of migratory progenitors constitutes a unique mode of neurogenesis in the *Drosophila* nervous system (Apitz and Salecker, 2015, 2018; Mora et al., 2018; Pinto-Teixeira et al., 2018). Recent work has shown an intricate network of signaling pathways and transcription factors that regulates the development of this region of the brain. Epithelial to mesenchyme transition (EMT) mediates the generation of migratory progenitors in the p-IPC neuroepithelium (Apitz and Salecker, 2015). These progenitors become neural stem cells (neuroblasts, NBs) upon arriving at the d-IPC. There, neuroblasts go through a temporal switch in transcription factor expression, from Asense (lower neuroblasts) to Atonal (upper neuroblasts), that allows the production of different sets of neurons (Apitz and Salecker, 2018; Mora et al., 2018; Oliva et al., 2014; Pinto-Teixeira et al., 2018). Thus, upper neuroblasts generate T4 and T5 neurons necessary for the processing of motion. T4/T5 neurons establish dendritic processes to get inputs from medulla and lobula neuropils, respectively (Figure 1A). Then, T4/T5 axonal terminals locate in one of the four layers of the lobula plate according to the direction of the visual stimuli.

Several signaling pathways involved in distinct steps of the differentiation of neurons of the motion detection system have been identified in the past few years (Apitz and Salecker, 2018; Mora et al., 2018; Pinto-Teixeira et al., 2018). However, less is known about the molecular bases regulating progenitor migration and morphogenesis of neuronal populations. The Slit-Robo pathway is a classic axon guidance regulator (Brose et al., 1999; Dickson and Gilestro, 2006; Kidd et al., 1999; Kidd et al., 1998) that, in the last years, has been implicated in several processes such as cell migration and cancer (Blockus and Chedotal, 2016). In *Drosophila* and vertebrates, the ligand Slit binds to different Robo receptors that regulate axon pathfinding decisions during development. In this context, Robo triggers a molecular cascade that modulates cytoskeleton behavior leading in most cases to neurite retraction (Dickson and Gilestro, 2006). In previous work, we and other groups have shown that Slit-Robo signaling is essential for correct boundary formation, morphogenesis and axon pathfinding in the *Drosophila* visual system. Slit secreted by neurons and glial cells is required for all these aspects (Caipo et al., 2020; Pappu et al., 2011; Suzuki et al., 2016; Suzuki et al., 2018; Tayler et al., 2004). While the function of Robo receptors is necessary for multiple cell populations (Pappu et al., 2011; Suzuki et al., 2016; Tayler et al., 2004), their contribution to specific regions of the visual system remains unclear.

In this work, we address the role of Slit and the three Robo receptors (Robo1, 2 and 3) during the development of the *Drosophila* lobula plate. We show that distinct combinations of the Robo receptors are expressed during different stages of lobula plate development. Robo2 is a key player in this process. Loss of function using a p-IPC specific driver leads to loss of cell stream integrity. Moreover, the combination of *robo1* and *robo2* knockdown produces an increase of cell adhesion molecules correlated with loss of stream integrity. Robo2 is also required for the correct structure of the neuroblast and neuron compartments in the d-IPC. Finally, Robo2 is necessary for the correct targeting of T4 dendrites in the medulla, a phenotype that is enhanced by *robo1* loss of function. We propose that during lobula plate development, the Slit-Robo signaling acts in every step from neuronal precursor migration to neuronal wiring.

## Results

### *slit* Mutants Exhibit Lobula Plate Structural Defects and Mistargeting of Motion Detection Neurons

*slit* mutant animals have been previously described to show defects in the adult optic lobe (Caipo et al., 2020; Pappu et al., 2011; Suzuki et al., 2016; Tayler et al., 2004). In these mutants, strong structural defects are present in the medulla, but the lobula plate is also affected (Caipo et al., 2020). In order to know whether specific lobula plate neurons are affected in these mutants, we focused on the T4 and T5 motion detection neurons (Maisak et al., 2013; Figure 1A). We expressed a membrane-tagged fluorescent protein (CD4-tdTomato) that highlights neuronal processes with great detail (Han et al., 2011), in combination with the *sli*^dui^ mutant allele, a hypomorphic allele generated by a p-element insertion that reduces *slit* expression (Pappu et al., 2011; Tayler et al., 2004). Using this tool, we observed defects in the targeting of T4 dendrites in *sli*^dui^ mutants (Figures 1B–E). T4 neurons reside in the lobula plate and receive inputs from neurons in the M10 layer of the medulla. In brains from *sli^dui^/* + animals, we found occasional mistargeting of T4 projections, however, in *sli*^dui^ mutants, T4 neurons overgrew to distal layers of the medulla in all scored animals and these ectopic projections were substantially thicker and probably reflecting axonal fasciculation (Figures 1C–F). Although these results indicate that Slit protein is necessary for the correct development of the lobula plate and T4 neurons in particular, it is unclear whether these defects are a direct result of the lack of Slit acting on Robo receptors in the lobula plate or a consequence of the role of Slit in medulla development.

### Slit and Robo Receptors Are Expressed During Lobula Plate Development

**FIGURE 2:**
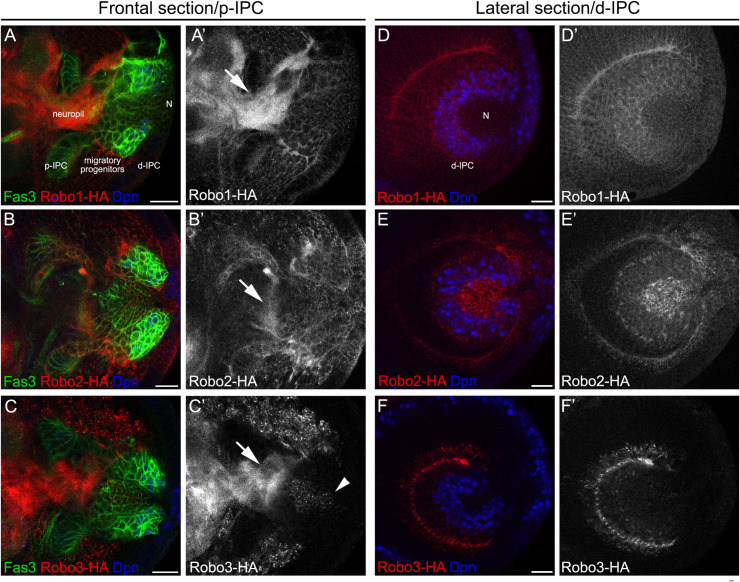
Differential expression of *robo* receptors in the larval optic lobe. **(A–C′)** Frontal and **(D–F′)** lateral sections of endogenously tagged Robo receptors. **(A,A′,D,D′)** *robo1-HA*, **(B,B′,E,E′)** *robo2-HA* and **(C,C′,F,F′)** *robo3-HA* larval brains stained against HA (red or gray), Fas3 (green) and Dpn (blue). d-IPC, distal inner proliferation center; p-IPC, proximal inner proliferation center; N, T4/T5 neurons. Arrowhead show T4/T5 cell bodies and arrows point Robo expression in the neuropil. Scale bars represent 20 μm.

To better understand the role of Slit-Robo signaling during lobula plate development, we analyzed the expression of the three Robo receptors and the Slit ligand in third instar larval stage. Two different anatomical views were examined; frontal sections allowed to observe all regions of the lobula plate precursors (p-IPC, cell streams and d-IPC), while lateral views permitted to observe the organization of the d-IPC in which stem cells form a horseshoe structure that surrounds the T4/T5 neurons that are displaced toward the center (see Figure 1A for a schematic representations). We used previously described knock-in lines of the Robo1-3 receptors tagged with a hemagglutinin epitope [HA, (Spitzweck et al., 2010)] in combination with the IPC marker Fasciclin3 (Fas3). We found that Robo1 and Robo2 are differentially expressed along the IPC; low expression is observed in the p-IPC neuroepithelium, while high expression is observed in migratory progenitors (Figures 2A–B′, see intensity quantifications in Supplementary Figure 1). Additionally, we observed that Robo1 and Robo2 were also expressed in neural stem cells (neuroblasts), labeled by Deadpan (Dpn), and early-born neurons located in the center of d-IPC (Figures 2D–E′). On the other hand, Robo3 is only expressed in cell bodies of more differentiated neurons and excluded from other domains of the IPC (see arrowhead in Figures 2C,C′,F,F′). Interestingly, all Robo receptors were found in the optic lobe neuropil (see arrows in Figures 2A′–C′).

Next, we analyzed the expression of Slit ligand using a previously described *slit:GFP* line [*sli^MI03825–GFSTF.2^*, (Caipo et al., 2020; Plazaola-Sasieta et al., 2019; Venken et al., 2011)]. We found that Slit was expressed in populations of glial cells that surround the p-IPC neuroepithelium and migratory progenitors (see arrowheads and arrow in Supplementary Figures 2G–I′″). We also observed low levels in cell streams and neuroblasts in the d-IPC, while an upregulation was observed in T4/T5 neurons (Supplementary Figures 2A–B′,G–I′″). This was confirmed using a *lacZ* insertion in the *sli* locus and an antibody against Slit protein (Supplementary Figures 2C–F′). These results show that during IPC development, both Robo1 and Robo2 are expressed and that there is a local source of Slit from glia and neurons.

### Requirement of Robo Receptors During Lobula Plate Development

To understand how Slit-Robo signaling regulates lobula plate development, we used a set of GAL4 drivers that allow gene expression manipulation in distinct stages of differentiation. We used *pIPC-GAL4* [R35B01, enhancer (Pfeiffer et al., 2008; Pinto-Teixeira et al., 2018)] driver for neuroepithelium and migratory progenitors; *insc-GAL4* and *wor-GAL4* for neuroblasts; an atonal enhancer *T4/T5-IPC-GAL4* (Oliva et al., 2014) and *acj6-GAL4* for developing T4/T5 neurons; and *bab2-GAL4* [R42F06, enhancer (Maisak et al., 2013; Pfeiffer et al., 2008)] to label adult T4/T5 neurons (Supplementary Figure 3).

**FIGURE 3:**
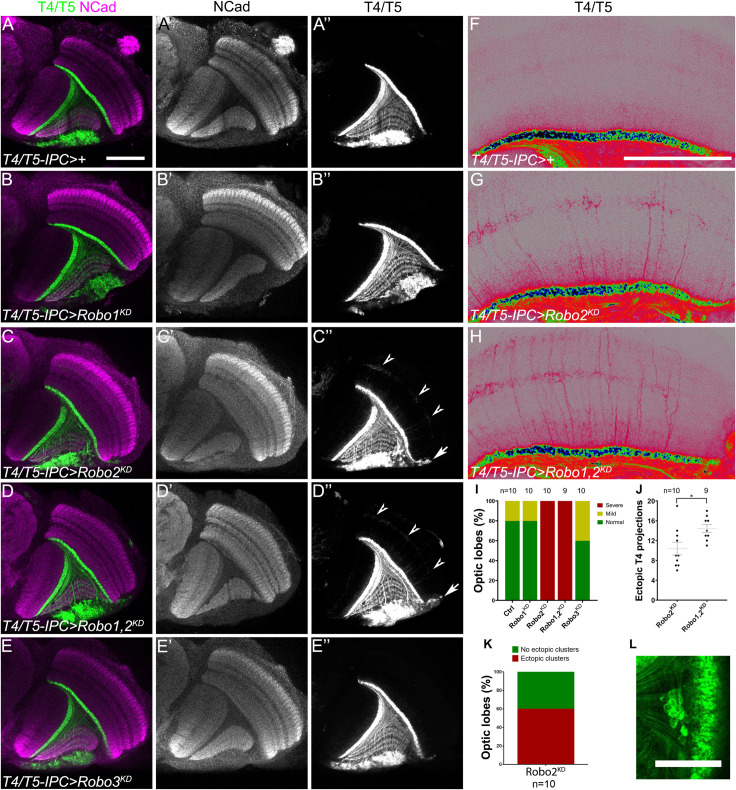
Robo2 is necessary in T4 neurons for dendrite targeting. **(A–H)** Horizontal sections of adult optic lobes of *T4/T5-IPC-GAL4, bab2-GAL4, UAS-CD4-tdTomato* line (*IPC* >) crossed to *w*^1118^ (Control), **(A–A″,F)**; *UAS-shRobo1* (Robo1^KD^), **(B–B″)**; *UAS-shRobo2* (Robo2^KD^), **(C–C″,G)**; *UAS-shRobo1* and *UAS-shRobo2*, (Robo1,2^KD^), **(D–D″,H)**, and *UAS-shRobo3* (Robo3^KD^), **(E–E″)**. Knockdown was driven by *T4/T5-IPC-GAL4* during development, while *bab2-GAL4* was used to mark adult T4/T5 neurons. Brains were stained for NCad (magenta or gray), and tdTomato (green, gray or pseudocolor) shows T4 and T5 neurites. Arrowheads point to ectopic T4 dendrites and arrows show ectopic T4/T5 cell bodies. **(F–H)** are amplified and pseudocolored images of panels **(A,C,D)**. **(I)** Bar chart showing the frequency of ectopic T4 projections for each genotype shown in panels **(A–E)**. **(J)** Quantification of the number of ectopic T4 dendrites for each genotype in equivalent optical sections. Mean and SEM are shown. Student’s *t*-test was performed. * is *p*-value = 0.0186. **(K)** Graph of the frequency of ectopic somas seen after *robo2* knockdown. **(L)** T4/T5 neuronal somas are ectopically found within the inner chiasm in *robo2* knockdown brains. Scale bars represent 50 μm in panels **(A–F)** and 25 in μm **(L)**.

Since *sli*^dui^ mutants displayed overgrowth of T4 dendrites, we wanted to determine whether this defect depends on Robo receptors being expressed in this population of neurons. To accomplish this, we used RNAi lines against each robo receptor that were able to significantly reduce their expression in neurons (see Supplementary Figure 4). RNAi-mediated knockdown of *robo1* and *robo3* in lobula plate neurons did not lead to defects in the targeting of T4 dendrites (Figures 3A–B″,E–E″,I). However, ectopic growth of T4 dendrites was observed upon knockdown of *robo2* (see arrowheads in Figures 3C–C″,G,I). Interestingly, this phenotype was enhanced after double knockdown of *robo1* and *robo2* (Robo1,2^KD^, Figures 3D–D″,H,J). Together these results suggest that both Robo1 and Robo2 are required in T4 neurons to restrict dendrite growth to the layer M10 of the medulla neuropil. Furthermore, we observed ectopic localization of lobula plate somas when *robo2* or *robo1* plus *robo2* were knocked down (see arrows in Figures 3C″,D″,K,L).

**FIGURE 4:**
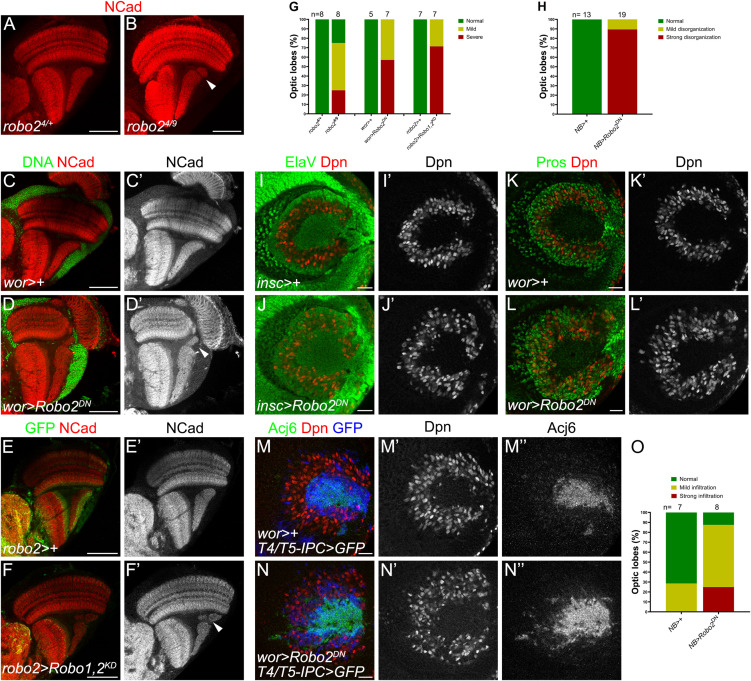
Robo2 in neuroblasts regulates lobula plate morphogenesis. **(A,B)** Immunostaining against NCad (red) of panels **(A)** *robo2^4/+^* heterozygous (Control) and **(B)** *robo2^4/9^* mutant adult optic lobes. **(C–D′)** Adult optic lobes of *wor-GAL4* (*wor* >) crossed to *w*^1118^ (Control), **(C,C′),** and *UAS-robo2*Δ*C* (Robo2^DN^), **(D,D′)**, and stained for NCad (red, gray) and DNA (green). **(E–F′)** Immunostaining of *robo2-GAL4, UAS-mCD8-GFP, robo1^1^* (*robo2* >) crossed to *w*^1118^ (Control), **(E,E′)** and *UAS-shRobo1, UAS-shRobo2* (Robo1,2^KD^) **(F,F′)**. Adult optic lobes were stained for GFP (green) and NCad (red, gray). Arrows point to disruptions in the lobula plate neuropil. **(G)** Bar chart showing the quantification of the phenotypes in panels **(A–F′)**. **(H)** Phenotype penetrance in larval optic lobe of each experimental condition **(I–L′)**. NB > is *wor* > or *insc*>. **(I–L)** Larval optic lobes of control **(I,I′,K,K′)** or Robo2^DN^ **(J,J′,L,L′)** expressed in neuroblasts using *insc-GAL4* (*insc* >), **(I–J′)** or *wor-GAL4* (*wor* >) **(K–L′)**, and stained for Dpn (red, gray), Elav (green) and Pros (green). **(M–N″)** Immunostaining against Acj6 (green, gray), Dpn (red, gray) and mCD8-GFP (blue) of *wor-GAL4, T4/T5-IPC-LexA, LexAop-mCD8-GF*P (*wor* > *IPC* > *GFP*) crossed to *w*^1118^ (Control), **(M–M″)** and *UAS-robo2*Δ*C* (Robo2^DN^), **(N–N″)**. **(O)** Penetrance of the ectopic progeny phenotype of animals in panels **(M–N″)**. Images of adult brains are horizontal sections and those of larval optic lobes are lateral sections. Scale bars represent 50 μm in panels **(A–F′)** and 20 μm in panels **(I–N″)**.

After knocking down *robo2* in neurons, we observed that although dendrites of T4 neurons were affected, the structure of the lobula plate neuropil was intact. Since *sli*^dui^ mutants have also defects in lobula plate morphology (Caipo et al., 2020), we hypothesized that structural defects may be due to Slit-Robo signaling deficiency in other cells of the IPC that would lead to morphological defects in the tissue. First, we analyzed adult optic lobes of *robo2* mutant animals, of a viable hypomorphic allele combination (*robo2^4/9^*), observing defects in the lobula plate neuropil architecture, while they presented a normal morphology in the medulla (Figures 4A,B,G). Next, we decided to determine whether loss of function of Robo2 specifically in IPC progenitors produced defects in the architecture of the lobula plate. We used the *wor-GAL4* driver line which is expressed in neuroblasts to determine if Robo2 is required in this context. In adult stage, we found structural defects in the lobula plate neuropil, upon expression of a dominant-negative form of Robo2 that lacks the cytoplasmic domain (Robo2^DN^) or double knocking down of *robo1* and *robo2* (Robo1,2^KD^) (Figures 4C–G). These defects were similar to those displayed in the *robo2* mutants. Interestingly, expression of Robo2^DN^ using T4/T5 neuronal drivers did not affect the morphology of the adult lobula plate neuropil, supporting a role of Robo2 in neural progenitors but not in neurons for the integrity of the adult neuropil (Supplementary Figure 5).

To understand the origin of these defects, we analyzed the organization of the d-IPC upon loss of Robo2 function in larval stage. In normal conditions, neuroblasts, marked by Dpn, are arranged in a horseshoe manner at the lateral side of the optic lobe, meanwhile the neuronal progeny of upper neuroblasts, marked by Elav, was extruded to the center (Figures 4I,I′″). We found that in Robo2^DN^ brains, the neuroblast domain is wider than in control conditions and Elav-positive progeny invaded the neuroblast domain (Figures 4H–J′). Using the GMC/neuron marker Prospero (Pros), we found that, unlike in control animals, Pros-positive cells remained next to neuroblasts (Figures 4K–L′). Similarly, this neuroblasts disorganization was observed in *sli*^dui^ mutant larval brains (see Supplementary Figure 6) as reported previously (Caipo et al., 2020), confirming that the architecture of the d-IPC requires Slit-Robo signaling.

The cells that invaded the neuroblast region could be T4/T5 neurons or the progeny of lower IPC neuroblasts [known as distal cells (Apitz and Salecker, 2018; Mora et al., 2018; Pinto-Teixeira et al., 2018)]. To determine whether T4/T5 neurons properly segregate from the neuroblast horseshoe region, we used Abnormal chemosensory jump 6 (Acj6) and *T4/T5-IPC-LexA, LexAop-mCD8-GFP* to label T4/T5 fate. In control optic lobes, we observed Acj6 and GFP at the center of the horseshoe, however, when neuroblasts expressed the Robo2^DN^, Acj6/GFP-positive T4/T5 neurons were localized among neuroblasts (Figures 4N,O). These results suggest that the structural defects observed in the adult lobula plate neuropil are caused by a lack of Robo receptor function in neuroblasts that is required for the proper segregation of neuroblasts, GMCs and T4/T5 neurons during larval development.

Given that Slit was highly expressed in T4/T5 neurons (Supplementary Figure 2) and Robo receptor function is needed in neuroblasts, we wondered whether Slit from neurons signals back to neuroblasts to maintain the segregation between neural stem cells and their progeny. Thus, we expressed an RNAi against *sli* in T4/T5 neurons using the *T4/T5-IPC-GAL4* driver in combination with a heterozygous background for the mutant allele *sli*^2^. We observed that in the knockdown of *sli*, T4/T5 neurons invaded the neuroblast field (see arrow in Supplementary Figure 7). This result supports the hypothesis that Slit from neuronal progeny activate Robo receptors in neuroblasts to maintain a proper organization in the d-IPC.

### Robo Signaling Is Required in IPC Migratory Progenitors for the Organization of the Lobula Plate

**FIGURE 5:**
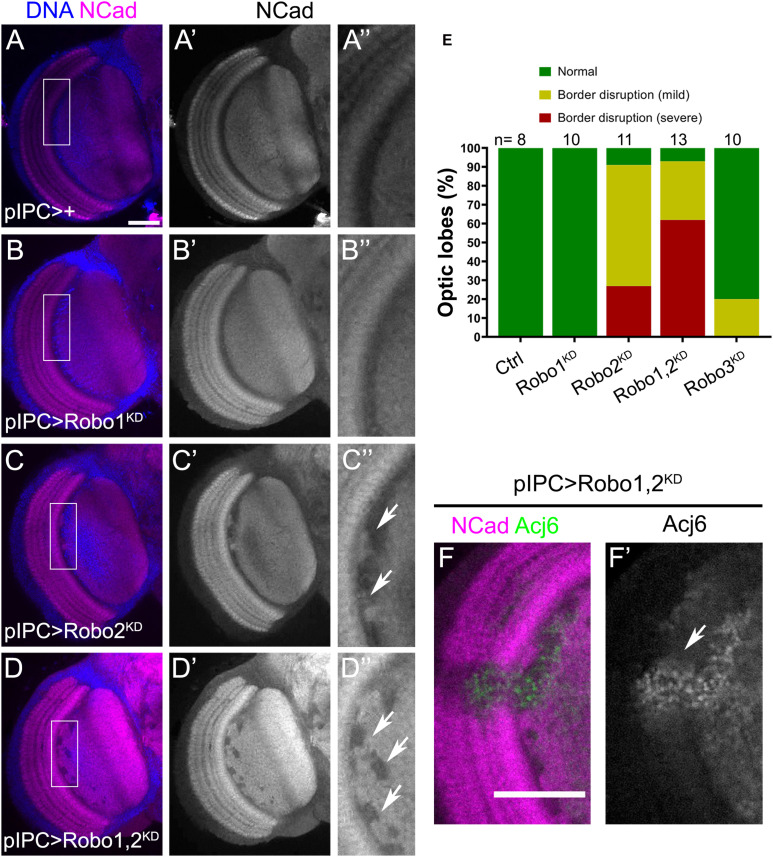
Migratory progenitors require Robo2 function for normal optic lobe development. **(A–D″)** Frontal sections of adult optic lobes stained for DNA (blue) and NCad (magenta, gray). Flies expressing *pIPC-GAL4* (*pIPC* >) were crossed to *w*^1118^ (Control), **(A–A″)**; *UAS-shRobo1* (Robo1^KD^) **(B–B″)**; *UAS-shRobo2* (Robo2^KD^), **(C–C″),** and *UAS-shRobo1*, *UAS-shRobo2* (Robo1,2^KD^), **(D–D″)**. Arrows point to neuropil defects in the lobula plate neuropil. **(E)** Graph showing the percentage of brains with morphological defects in panels **(A–D″)**. **(A″,B″,C″,D″)** are magnifications of the rectangular region in panels **(A–D)**. Fisher’s exact tests was performed, Robo2^KD^ compared to Robo1,2^KD^ has an adjusted *p*-value = 0.364. **(F,F′)** Ectopic Acj6 positive cells (arrow) in fly brains expressing *UAS-shRobo1*, *UAS-shRobo2* (Robo1,2^KD^) under the control of the *pIPC-GAL4* driver. Adult optic lobes stained for NCad (magenta) and Acj6 (green, gray). Scale bars represent 50 μm in panels **(A–D,F,F′)**.

Our expression analysis showed that Robo1 and Robo2 are highly expressed in migratory progenitors of the lobula plate (Figures 2A–B′). To find whether Robo2 is needed in this cell population for lobula plate development we used *pIPC-GAL4* (Pinto-Teixeira et al., 2018) to reduce *robo1* and *robo2* expression. Expressing RNAi against *robo1* did not affect the morphology of the lobula plate neuropil (Figures 5A–B″), however, in *robo2* knockdown we observed defects in lobula plate neuropil architecture of adult animals (Figures 5C–C″). Although, the severity of these defects appeared to be increased after double knockdown of *robo1* and *robo2*, this was not statistically significant (Figures 5D,E). In this condition, we also observed an invasion of T4/T5 neurons, marked by Acj6, in the medulla neuropil (Figures 5F,F′). These results suggest that in the IPC neuroepithelium and migratory progenitors, Robo2 is necessary for proper lobula plate development.

**FIGURE 6:**
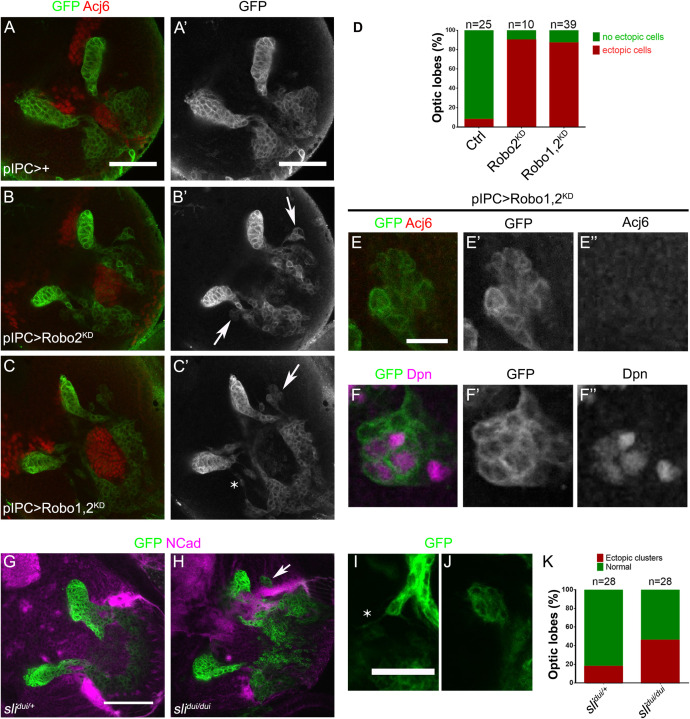
Robo2 function is necessary for proper migration of progenitors during development. **(A–C′)** Frontal sections of larval optic lobes of *pIPC-GAL4, UAS-mCD8-GFP* animals crossed to *w*^1118^ (Control), **(A,A′)**; *UAS-shRobo2* (Robo2^KD^), **(B,B′),** and *UAS-shRobo1*, *UAS-shRobo2* (Robo1,2^KD^), **(C,C′)**, and stained for GFP (green, gray) and Acj6 (red). Arrows show ectopic clusters of migratory progenitors. **(D)** Graph showing the frequency of ectopic clusters in panels **(A–C′)**. **(E-F″)** Ectopic clusters of migratory progenitors observed after knocking down both *robo1* and *robo2* (Robo1,2^KD^), stained for GFP (green, gray), Acj6 (red, gray) and Dpn (magenta, gray). **(G,H)** Larval optic lobes of panels **(G)** *sli*^dui/+^ (Control) and **(H)** *sli*^dui/dui^ mutant animals expressing *UAS-mCD8-GFP* (green) under the control of *pIPC-GAL4* and stained for NCad (magenta). Arrow points to an ectopic cluster. **(I,J)** Ectopic clusters of migratory progenitors observed in *sli*^dui/dui^ mutant animals. Asterisk shows a cell extending a protrusion outside the migratory stream. **(K)** Graph showing the frequency of presence of ectopic clusters of migratory progenitors in panels **(G,H)**. Scale bars represent 50 μm in panels **(A–C′,G,H)** and 25 μm in panels **(E–F″,I,J)**.

To understand how this phenotype is generated during development, we analyzed defects in the larval brain. In *robo2* knockdown animals, neuroepithelial cells of the p-IPC showed normal morphology compared to control brains, however, we found ectopic clusters of cells connected with the streams of migratory progenitors (see arrows in Figures 6A–B′). When both *robo1* and *robo2* were knocked down, we observed a similar number of ectopic clusters compared to *robo2* knockdown condition (Figures 6C,D). These clusters could also be observed in *sli*^dui^ mutant larval brains (Figures 6G–K). Interestingly, these ectopic clusters contained some cells that expressed the neuroblast marker Dpn, but did not express the T4/T5 neuron marker Acj6 (Figures 6E–F″). These results suggest that the Slit-Robo signaling, mainly through the function of Robo2, is required for the correct migration and positioning of migratory progenitors.

### Ectopic Clusters of Migratory Progenitors Show Increased Expression of Adhesion Molecules

The Slit-Robo signaling pathway can regulate numerous cellular processes. The best described downstream factors involved in neuronal development are cytoskeleton regulators (Blockus and Chedotal, 2016). However, more recently it has been shown that Robo can also regulate cell adhesion in certain cellular contexts (Santiago-Martinez et al., 2008; Vaughen and Igaki, 2016). Interestingly, in the IPC context, we find that the level of Robo2 expression is increased in migratory progenitors (Supplementary Figure 1E), while it has been reported that the expression of E-Cadherin (ECad) decreases in these cells compared to the neuroepithelium (Apitz and Salecker, 2015).

**FIGURE 7:**
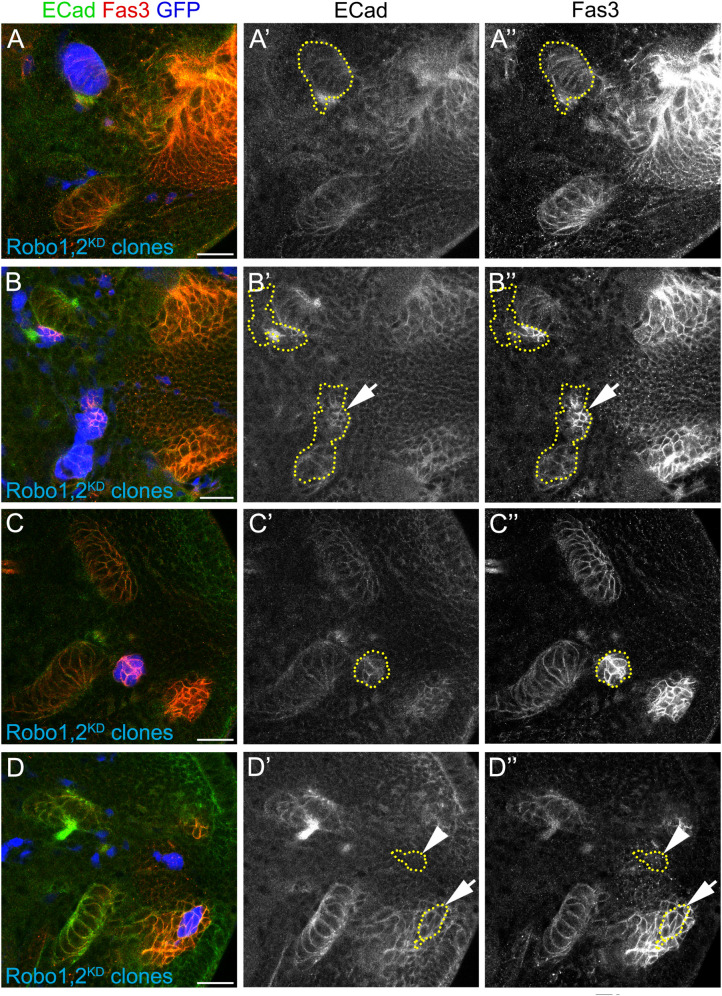
Ectopic clusters upregulate adhesion molecules. **(A–D″)** Larval optic lobes with clones expressing *UAS-shRobo1*, *UAS-shRobo2* (Robo1,2^KD^) and marked by UAS-GFP (blue), were stained for ECad (green, gray) and Fas3 (red, gray). Clones at different stages of the IPC differentiation are shown. **(A–A″)** p-IPC neuroepithelial clone, **(B–B″)** p-IPC neuroepithelial/migratory progenitor clone (arrows), **(C–C″)** migratory progenitor clone, **(D–D″)** neuroblast (arrows), and neuronal clones (arrowhead). Number of clones analyzed was 8 for neuroepithelia, 10 for migratory progenitors, 3 for neuroblasts and 7 for neurons. 100% of the clones in migratory progenitors presented an increase in ECad/Fas3 expression. Scale bars represent 20 μm. For control clones, see Supplementary Figure 8.

To understand how migratory progenitors are affected after the loss of the Slit-Robo signaling, we generated flip-out clones expressing RNAi against *robo1* and *robo2*, and analyzed the levels of the adhesion molecules Fas3 and ECad (control clones can be seen in Supplementary Figure 8). We found that both adhesion molecules were highly expressed in the neuroepithelium but not migratory progenitors, as described before (Apitz and Salecker, 2015). Similar to the knockdown using *pIPC-GAL4* (Figures 6A–C′), *robo1* and *robo2* knockdown clones in the neuroepithelium did not affect its morphology, nor the expression of ECad and Fas3 (Figures 7A–A″). However, when these clones left the neuroepithelium we observed an increase in the expression of Fas3 and ECad (see arrows in Figures 7B–B″). Strikingly, this adhesion molecule upregulation is only observed in ectopic clusters in the migratory progenitor region, and clones seemed to lose their normal collective behavior (Figures 7C–C″). Finally, when *robo1* and *robo2* knockdown clones were generated in the neuroblast region or in T4/T5 neurons, we did not observe a clustering phenotype or changes in the levels of ECad and Fas3 (see arrows and arrowheads in Figures 7D–D″). Altogether, these results support a model in which the Slit-Robo signaling downregulates the levels of adhesion molecules to promote the integrity of migratory progenitors.

## Discussion

**FIGURE 8:**
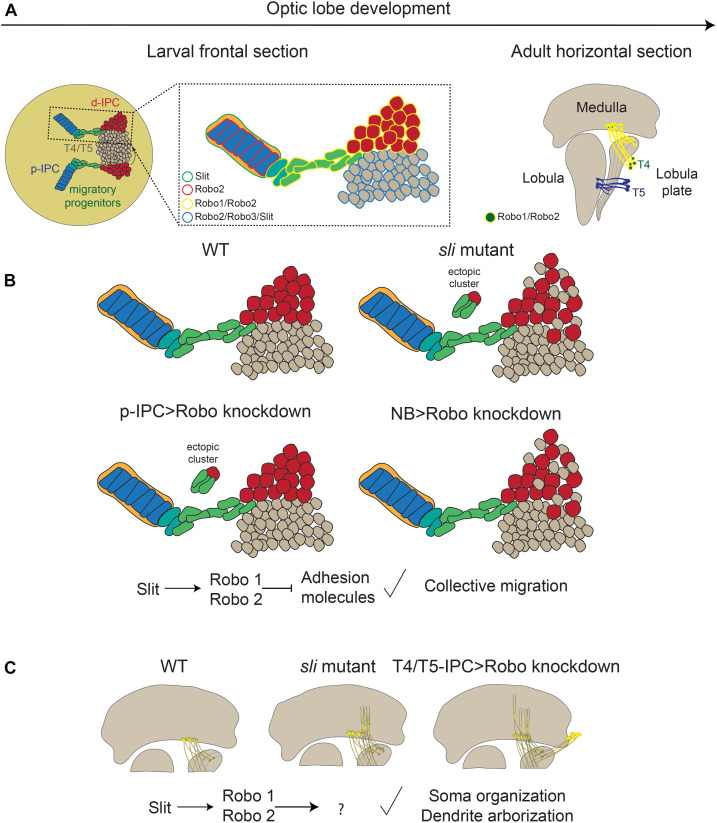
The role of the Slit/Robo signaling pathway during the differentiation of motion detection neurons. **(A)** Expression of the components of the Slit/Robo pathway during optic lobe development. **(B)** Phenotypes observed in *sli*t mutant and loss of *robo* receptors in migratory progenitors and neuroblasts. **(C)** Loss of Slit/Robo signaling during T4 neuron differentiation affects the dendrite targeting in the medulla and the localization of the soma. d-IPC, distal inner proliferation center; p-IPC, proximal inner proliferation center; T4/T5, T4, and T5 motion detection neurons.

Classic axon guidance signaling pathways have been shown to control additional cellular processes, including proliferation and adhesion, and therefore regulating tissue morphogenesis. In this work we show that the Slit-Robo pathway is involved in the development of the lobula plate, playing specific roles in different stages of neural differentiation. Thus, Robo2 controls neuroepithelial adhesion and progenitor migration, neuroblast compartment organization and the proper targeting of dendrites of motion detection neurons (Figure 8).

### Slit-Robo Signaling Controls the Wiring of T4 Neurons

The mechanisms controlling the wiring of motion detection neurons in the *Drosophila* visual system, have only recently received attention. First, it was shown that the function of the transcription Atonal, a known proneural gene, impacts the wiring of these neurons in an indirect manner since it is expressed in neural stem cells but not in differentiated neurons (Apitz and Salecker, 2018; Mora et al., 2018; Oliva et al., 2014; Pinto-Teixeira et al., 2018). Further work has revealed that a network of transcription factors is involved in the control of the targeting of T4 and T5 dendrites in the M10 layer of the medulla and the L1 layer of the lobula, respectively. This network is composed by Ato, and the transcription factors of the SRY Box family, SoxNeuro (SoxN) and Sox102F/SoxD (Contreras et al., 2018; Schilling et al., 2019). Loss of function of SoxN and SoxD leads to an overgrowth of T4 and T5 dendrites (Contreras et al., 2018; Schilling et al., 2019). Furthermore, loss of SoxD results in increased levels of the adhesion molecule Connectin. However, it is unclear whether Connectin plays a role in T4/T5 dendrite guidance, since knockdown of *connectin* cannot rescue the loss of function of SoxD (Schilling et al., 2019).

Two recent articles showed the transcriptional profiling during several stages of pupal development of all eight subtypes of T4/T5 neurons (named a, b, c and d), showing that a specific combination of transcription factors define the identity of each subtype (Hormann et al., 2020; Kurmangaliyev et al., 2019). Kurmangaliyev et al., using a single-cell RNA sequencing, found an enrichment of Robo2 in T4/T5 neurons of the c and d types. We showed T4 neurite targeting defects upon robo2 knockdown, which were enhanced by robo1 knockdown. Although we propose this is produced by an overgrowth of T4 dendrites, we cannot discard a function in axonal formation. Given that we did not find defects in T5 neurite targeting in our experiments, we believe that other guidance receptors may be responsible for restricting the growth of these dendrites. Interestingly, Hormann et al. found an enrichment of Robo3 in T4/T5c, d neurons, suggesting that Robo2 and Robo3 may act in these neurons in a redundant manner. In our experiments, we did not find defects upon expression of RNAi against *robo1* or *robo3*, however, we do not exclude the possibility that this is consequence of inefficient knockdown or the driver used are only strong during early stages of neuronal differentiation.

How transcription factor networks, which define T4/T5 identity, regulate the Robo-Slit signaling is unknown. It is plausible that the Robo-Slit signaling is downstream of the T4/T5 fate decision, and the phenotype observed in our experiments only obeys to a dendrite guidance defect.

### Slit-Robo in the Organization of the d-IPC

In the d-IPC, neural stem cells are arranged in a particular compartment forming a horseshoe shape. Two related populations of neuroblasts are present. While Asense-expressing neuroblasts (lower) give rise to distal cells that locate at the periphery of the horseshoe, Atonal-expressing neuroblasts (upper) produce T4 and T5 neurons (Apitz and Salecker, 2015, 2018; Mora et al., 2018; Oliva et al., 2014; Pinto-Teixeira et al., 2018). Upon birth, undifferentiated T4/T5 neurons are displaced to the center of the horseshoe. In *sli* mutants, neurons and stem cells intermingle at the interface between the two domains, indicating a cell segregation defect (Caipo et al., 2020). Defects in cell segregation have been also described in *robo2* and *robo3* mutant optic lobes (Suzuki et al., 2016), however, individual populations in which Robo receptors played a role have not been identified using targeted loss of function approaches. Here we observed that signal transduction by Robo is necessary in d-IPC neuroblast and Slit is required in neurons for the correct segregation of these two populations. The mechanisms involved may be associated to contact inhibition of locomotion, in which the Slit-Robo pathway has a known role (Fritz et al., 2015). However, the cells that are segregated are those expressing Slit, and our data indicate that neurons also express Robo2 and Robo3. It is possible that other pathways control the cytoskeletal dynamics of these neurons to allow leaving the neuroblast cluster, or the combination of different Robo receptors in these populations generates a differential collective behavior. Additional work will shed light on this aspect of the IPC development.

### Slit-Robo in the Integrity of Streams of Migratory Progenitors

Recent research has described the development of the lobula plate and the presence of a migratory stage that makes the transition between neuroepithelial cells and neuroblasts. This process involves epithelial to mesenchymal transition (EMT) from the p-IPC neuroepithelium (Apitz and Salecker, 2015). Strikingly, a known factor involved in this process in *Drosophila*, the Snail-like protein Escargot, is also a key regulator of EMT in this context. Previous work has shown that the downregulation of the adhesion molecule ECad is necessary for the correct formation of the stream of migratory cells in the d-IPC. Escargot is necessary to decrease ECad levels, since upon *escargot* knockdown, ectopic clusters of cells bearing high levels of ECad were observed connected to the neuroepithelium (Apitz and Salecker, 2015). We found that the levels of Robo2 are higher in migrating progenitors compared with the neuroepithelium, indicating a negative correlation with ECad levels. It has been demonstrated that ECad is downregulated by Robo in cardioblast during the formation of the *Drosophila* heart (Santiago-Martinez et al., 2008). Likewise, Slit and Robo2 participate in cell extrusion of tumorigenic cells, which is also mediated by decreasing the levels of ECad in the *Drosophila* epithelia (Vaughen and Igaki, 2016). Furthermore, Slit2 and Robo1 promote degradation of ECad and expression of EMT markers in human colorectal carcinoma cell lines (Zhou et al., 2011), suggesting that this regulation is conserved between flies and mammals. In contrast, defects produced by Robo2 loss of function in cyst stem cells of the *Drosophila* testis can be rescued by overexpression of ECad (Stine et al., 2014) indicating that the nature of the regulation depends on the cellular context. Thus, several lines of evidence support a regulation of ECad by Robo proteins, which is consistent with our observations. It is worth noting that this regulation is only observed in migratory progenitors, indicating that some other mediators are responsible of the Slit and Robo phenotypes observed in other regions of the IPC.

We postulate that Slit is broadly present in the developing *Drosophila* optic lobe, however, depending on the stage of differentiation, from neuroepithelial cells to neurons, different targets act downstream of the Slit-Robo signaling. The distinct role of the Slit-Robo signaling in different cell types may be explained by the expression of combinations of Robo receptors and the possible interaction with other pathways.

## Materials and Methods

### Fly Husbandry

Flies were kept at 25°C on standard medium. RNAi-mediated knockdown experiments were performed at 29°C. The GAL4 system (Brand and Perrimon, 1993) was used for RNAi and overexpression experiments. The lines used in our study were obtained from the Bloomington *Drosophila* Stock Center (BDSC, Bloomington, Indiana). The following mutant alleles were used: *sli^dui/dui^, GMR-GFP* [(Tayler et al., 2004), BDSC #9284], *sli*^2^ [(Nusslein-Volhard et al., 1984; Rothberg et al., 1988], BDSC#3266), *robo1*^1^ [(Kidd et al., 1998), BDSC #8755], *robo2*^4^ [(Rajagopalan et al., 2000), BDSC #66884] and *robo2*^9^ [(Rajagopalan et al., 2000), BDSC #66881]. *w*^1118^ was used as control. For characterizing endogenous gene expression we used *robo1-HA*, *robo2-HA* and *robo3-HA* (Spitzweck et al., 2010); *sli:lacZ* [(Spradling et al., 1999), BDSC #12189] and *sli:GFP* (*sli^MI03825–GFSTF.2^*, BDSC # 64472). We used the GAL4 drivers: *T4/T5-IPC-GAL4* (Oliva et al., 2014), *wor-GAL4* (Albertson et al., 2004), *insc-GAL4* (Luo et al., 1994), *pIPC-GAL4* (R35B01, BDSC #49898), *bab2-GAL4* (R42F06, BDSC #41253), *acj6-GAL4* (BDSC #30025), *robo2-GAL4* (DGRC #112604), *repo-GAL4* (Sepp et al., 2001). For knockdown experiments we used *UAS-Dicer2* (Dietzl et al., 2007), *UAS-shRobo1* (BDSC #39027), *UAS-shRobo2* (BDSC #34589), *UAS-shRobo3* (BDSC #44539), *UAS-Sli-RNAi* (BDSC #31468) and *UAS-Robo2*Δ*C* (Kraut and Zinn, 2004). *UAS-GFP*, *UAS-mCD8-GFP*, *UAS-mCD8-RFP*, *UAS-CD4-tdTomato* were used to mark driver expression.

Flip-out clones were generated using *y,w, hsFLP; AyGAL4, UAS-GFP/Cy*O and giving a 10 min heat shock at 37°C, 24 or 48 hrs. before dissection.

### Staining

Third instar larval and adult brains were dissected and stained using standard procedures (Contreras et al., 2018; Wu and Luo, 2006). Briefly, brains were dissected and fixed in 4% formaldehyde in 1X PBS for 20 min at room temperature. Samples were washed three times in PBT (0.3% Triton X-100 in 1X PBS) and blocked in 1% BSA/PBT for 30 min at room temperature. Antibodies were diluted in PBT and samples were incubated overnight at 4°C. The following monoclonal antibodies were obtained from Developmental Studies Hybridoma Bank: rat anti-N-Cadherin (DN-Ex #8; 1:20), rat anti-DE-Cadherin (DCAD2; 1:20), rat anti-Elav (7E8A10, 1:20), mouse anti-Slit (C555.6D; 1:50), mouse anti-Acj6 (1:10), mouse anti-Fasciclin3 (7G10; 1:20), mouse anti-Pros (MR1A, 1:20). Other antibodies used were guinea pig anti-Dpn (1:5,000, kind gift from Dr. Andrea Brand), rabbit anti-HA (C29F4, Cell Signaling, 1:500) and rabbit anti-GFP (A11122, Invitrogen, 1:1,000). Fluorescent-dye conjugated secondary antibodies were obtained from Jackson Immunoresearch and used 1:200. Hoechst was used as DNA counterstain (1:1,000).

### Imaging

Images were obtained using an Olympus Fluoview-Fv1000, Leica SP8X, Zeiss 700, Zeiss LSM 880 with Airyscan and Nikon Ti2-E confocal microscopes. All images were processed with Image J software (NIH) and the montage of figures were performed using Adobe Photoshop CC. Schemes were designed using Adobe Illustrator CS3.

### Data Analysis and Quantifications

Data analysis and graphs were done with GraphPad Prism 8 software. Two-tailed Student’s *t*-test was used. Data were presented as phenotype frequency or mean ± standard error of the mean. Phenotype severity was evaluated following examples shown in Supplementary Figure 9.

## Data Availability Statement

The raw data supporting the conclusions of this article will be made available by the authors, without undue reservation.

## Author Contributions

PG-P, EC, and CO designed the research, analyzed the data, and wrote the manuscript. JC, JS, and BH contributed to the expertise and reagents. PG-P, EC, NM, CG-R, and MS performed experiments. All authors contributed to the article and approved the submitted version.

## Conflict of Interest

The authors declare that the research was conducted in the absence of any commercial or financial relationships that could be construed as a potential conflict of interest.

## Supplementary Material

Supplementary Figure 1 Robo expression during IPC differentiation. **(A–C′)** Frontal sections of endogenously tagged Robo receptors. **(A–A″)** *robo1-HA*, **(B–B″)** *robo2-HA* and **(C–C″)** *robo3-HA* animals stained against HA (magenta. pseudocolor) and Fas3 (green). Arrows point to migratory progenitors. **(D–F)** Intensity analysis of HA-tagged Robo receptors across the white dashed line in panels **(A–C)** respectively. d-IPC, distal inner proliferation center; p-IPC, proximal inner proliferation center. Scale bar represents 50 μm.

Supplementary Figure 2 Slit expression in the IPC. **(A–F′)** Lateral sections of larval brains immunostained against Slit (green, gray) and Dpn (red). **(A–B′)** *slit:GFP* with anti-GFP antibody, **(C–D′)** *slit-LacZ* with anti-βGAL antibody, and **(E–F′)** *w*^1118^ using anti-Slit antibody. **(G–H′)** Frontal section of *slit:GFP* (green) larval optic lobes stained for Repo (red, glial marker). **(I–I′″)** Frontal sections of *slit:GFP* (green), *repo-GAL4, UAS-mCD8-RFP* (red, glial membrane marker) larval optic lobe. DNA is stained in blue. See co-localization between Slit and glial membrane in the p-IPC. d-IPC, distal inner proliferation center; p-IPC, proximal inner proliferation center; MP, migratory progenitors; N, T4/T5 neurons. Arrowhead show glial cell membrane covering the p-IPC, and arrow show migratory progenitors. Scale bars represent 20 μm.

Supplementary Figure 3 Expression of GAL4 drivers in the larval IPC. **(A–F″)** Larval optic lobes stained for GFP (green, gray), Dpn (red, gray) and Elav (blue), using different GAL4 drivers to target all the cell populations in the IPC. **(A–A″)** *robo2-GAL4*, **(B–B″)** *insc-GAL4*, **(C–C″)** *wor-GAL4*, **(D–D″)** *T4/T5-IPC-GAL4*, **(E–E″)** *acj6-GAL4*, and **(F–F″)** *pIPC-GAL4*. **(A–E″)** Lateral and **(F–F″)** frontal sections. Scale bars represent 20 μm.

Supplementary Figure 4 Robo knockdown efficiency. Larval optic lobes stained for **(A–B″)** Robo1, **(D–E′)** Robo2-HA and **(G–H′)** Robo3. *elav-GAL4* animals were crossed to **(A,A′,D,D′,G,G′)** *w*^1118^ (control), **(B,B′)** *UAS-shRobo1* (Robo1^KD^), **(E,E′)** *UAS-shRobo2* (Robo2^KD^) and **(G,G′)** *UAS-shRobo3* (Robo3^KD^). **(A′,B′,D′,E′,G′,H′)** are pseudocolored images of Robo intensity. Scale bars represent 50 μm. **(C,F,I)** Graphs showing intensity quantifications of Robo1, Robo2 and Robo3 signal in control and knockdown conditions. Student’s *t*-test was performed. *P*-value are **(C)** 3.59 × 10^−4^, **(F)** 3.26 × 10^−4^, **(I)** 0.0189.

Supplementary Figure 5 Loss of Robo2 in neurons does not affect the morphology of the lobula plate. **(A–D)** Horizontal sections of adult optic lobes stained against GFP (green) and NCad (red). **(A,B)** *T4/T5-IPC-GAL4* and **(C,D)** *acj6-GAL4* crossed to panels **(A,C)** *w*^1118^ and **(B,D)** *UAS-robo2*Δ*C* (Robo2^DN^). **(E)** Quantification of the frequency of morphological defects in the lobula plate. Scale bars represent 50 μm.

Supplementary Figure 6 d-IPC architecture is affected in *sli* mutant animals. **(A–B′)** Distal sections of larval optic lobes of panels **(A,A′)** *w*^1118^ and **(B,B′)** *sli^dui/dui^, GMR-GFP* animals. Brains were stained for GFP (green), Dpn (red, gray) and Fas3 (blue). **(C)** Graph showing the frequency of disruption in the organization of the d-IPC neuroblasts. Scale bars represent 20 μm.

Supplementary Figure 7 Slit is required in T4/T5 neurons for neuroblast organization. **(A–B″)** Lateral sections of larval optic lobes stained against GFP (green), Dpn (red, gray) and Slit (blue, pseudocolored). *T4/T5-IPC-GAL4, UAS-Dicer2, UAS-Stinger* were crossed to panels **(A,A″)** *w*^1118^ and **(B,B″)** *sli*^2/+^, *UAS-Sli-RNAi*. Arrow shows GFP-positive neurons invading the neuroblasts horseshoe region. **(C)** Graph showing the frequency of brains containing neurons mislocalized in the neuroblast region. Scale bars represent 20 μm.

Supplementary Figure 8 Wild type clones. **(A–D″)** Larval optic lobes with control clones marked by UAS-GFP (blue), were stained for ECad (green, gray) and Fas3 (red, gray). Clones at different stages of the IPC differentiation are shown. **(A–A″)** p-IPC neuroepithelial/migratory progenitor clone p-IPC neuroepithelial clone, **(B–B″)** neuroblast clone, **(C–C″)** neuroblast/neuronal clone, **(D–D″)** p-IPC neuroepithelial. No changes in Fas3 or ECad levels were observed. Scale bars represent 20 μm.

Supplementary Figure 9 Examples of phenotypes. Reference images used to determine the penetrance of the phenotypes in different experiments.
