# Supplementary figures and images for: Slit/Robo Signaling Regulates Multiple Stages of the Development of the Drosophila Motion Detection System
Source: Front Cell Dev Biol. 2021 Apr 21;9:612645. doi: 10.3389/fcell.2021.612645 (PMC8097104; doi:10.3389/fcell.2021.612645)

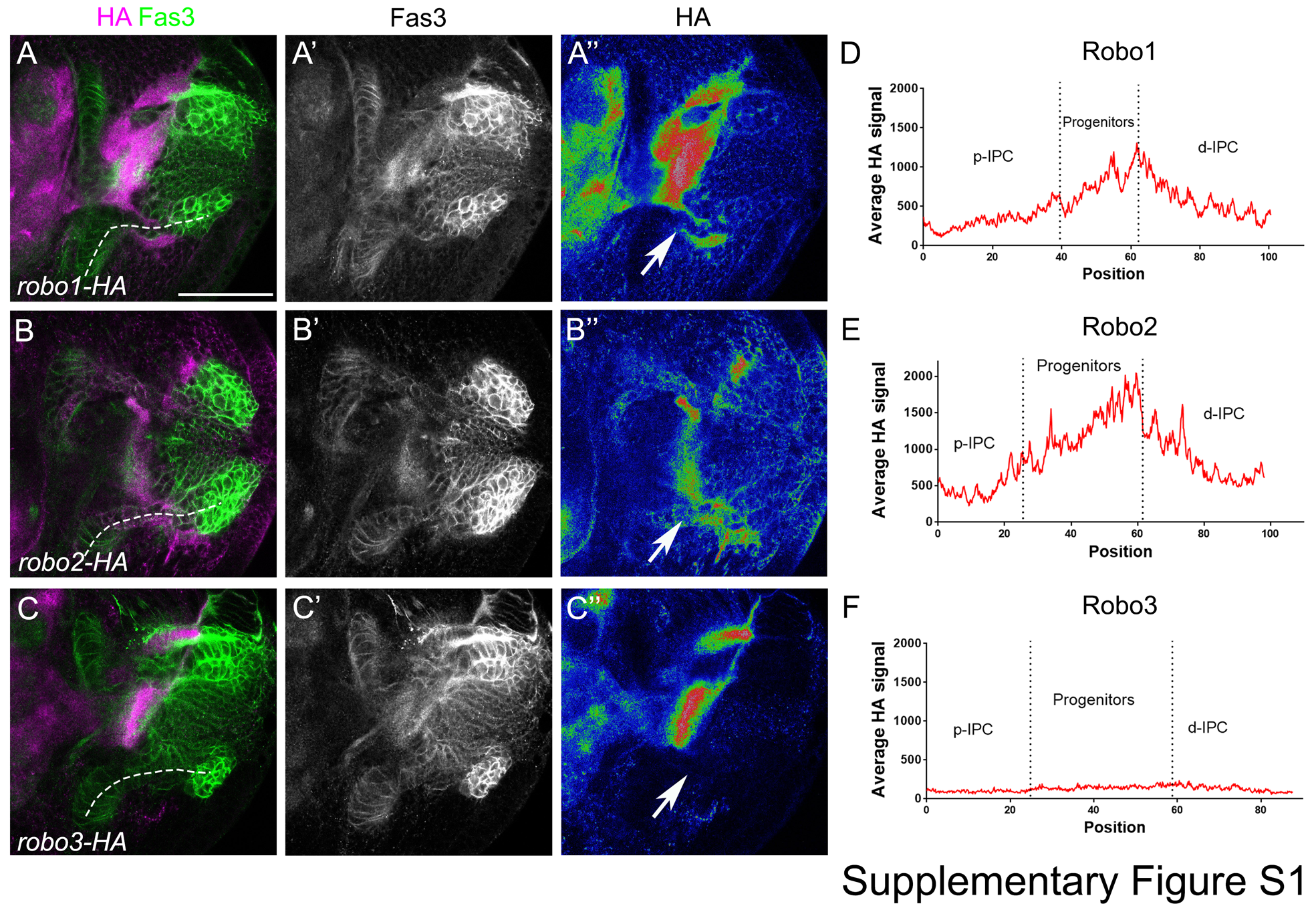

Supplement: Supplementary Figure 1 — Robo expression during IPC differentiation. (A–C′) Frontal sections of endogenously tagged Robo receptors. (A–A″) robo1-HA, (B–B″) robo2-HA and (C–C″) robo3-HA animals stained against HA (magenta. pseudocolor) and Fas3 (green). Arrows point to migratory progenitors. (D–F) Intensity analysis of HA-tagged Robo receptors across the white dashed line in panels (A–C) respectively. d-IPC, distal inner proliferation center; p-IPC, proximal inner proliferation center. Scale bar represents 50 μm. [file Image_1.TIF]

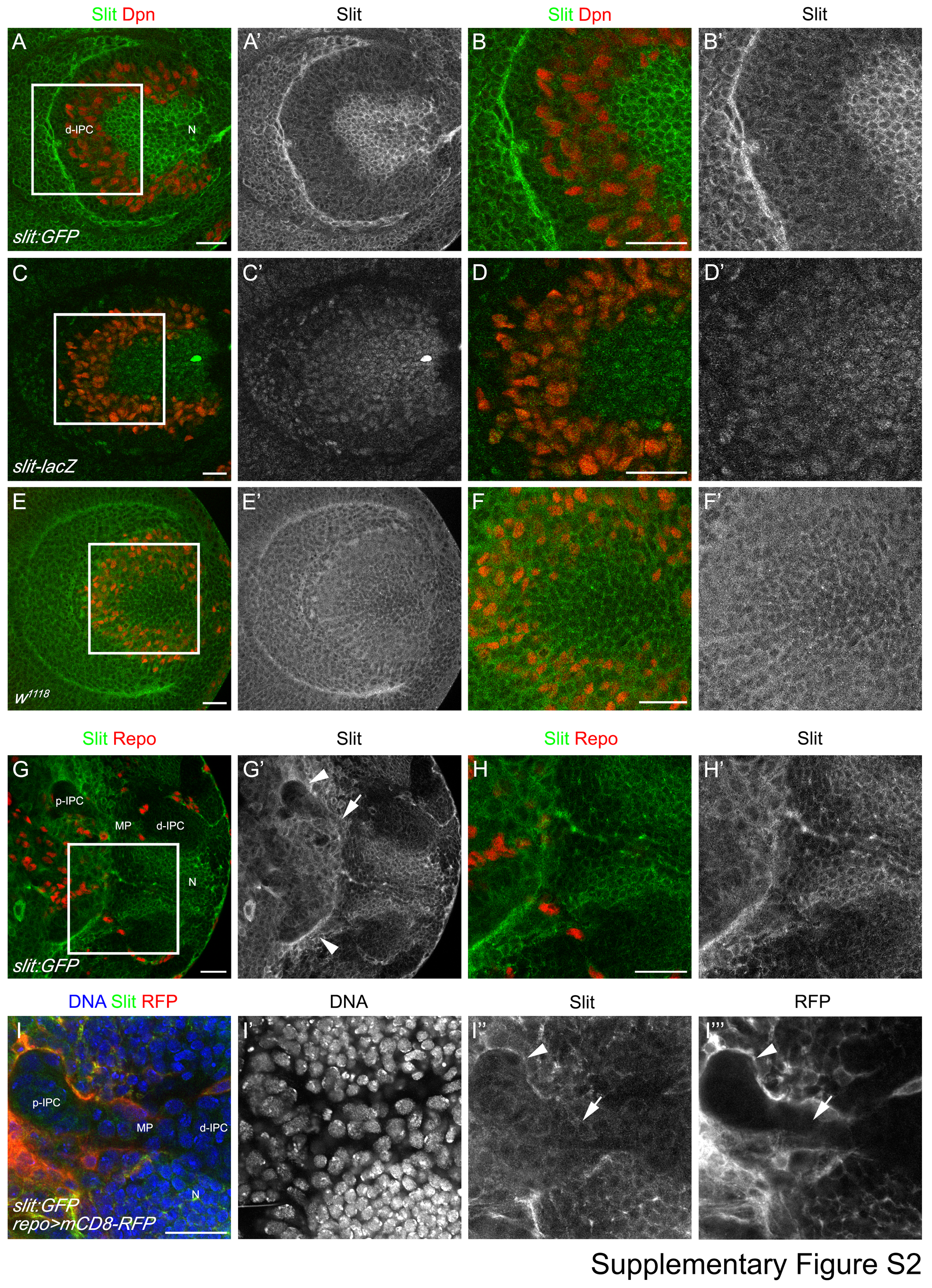

Supplement: Supplementary Figure 2 — Slit expression in the IPC. (A–F′) Lateral sections of larval brains immunostained against Slit (green, gray) and Dpn (red). (A–B′) slit:GFP with anti-GFP antibody, (C–D′) slit-LacZ with anti-βGAL antibody, and (E–F′) w1118 using anti-Slit antibody. (G–H′) Frontal section of slit:GFP (green) larval optic lobes stained for Repo (red, glial marker). (I–I′″) Frontal sections of slit:GFP (green), repo-GAL4, UAS-mCD8-RFP (red, glial membrane marker) larval optic lobe. DNA is stained in blue. See co-localization between Slit and glial membrane in the p-IPC. d-IPC, distal inner proliferation center; p-IPC, proximal inner proliferation center; MP, migratory progenitors; N, T4/T5 neurons. Arrowhead show glial cell membrane covering the p-IPC, and arrow show migratory progenitors. Scale bars represent 20 μm. [file Image_2.TIF]

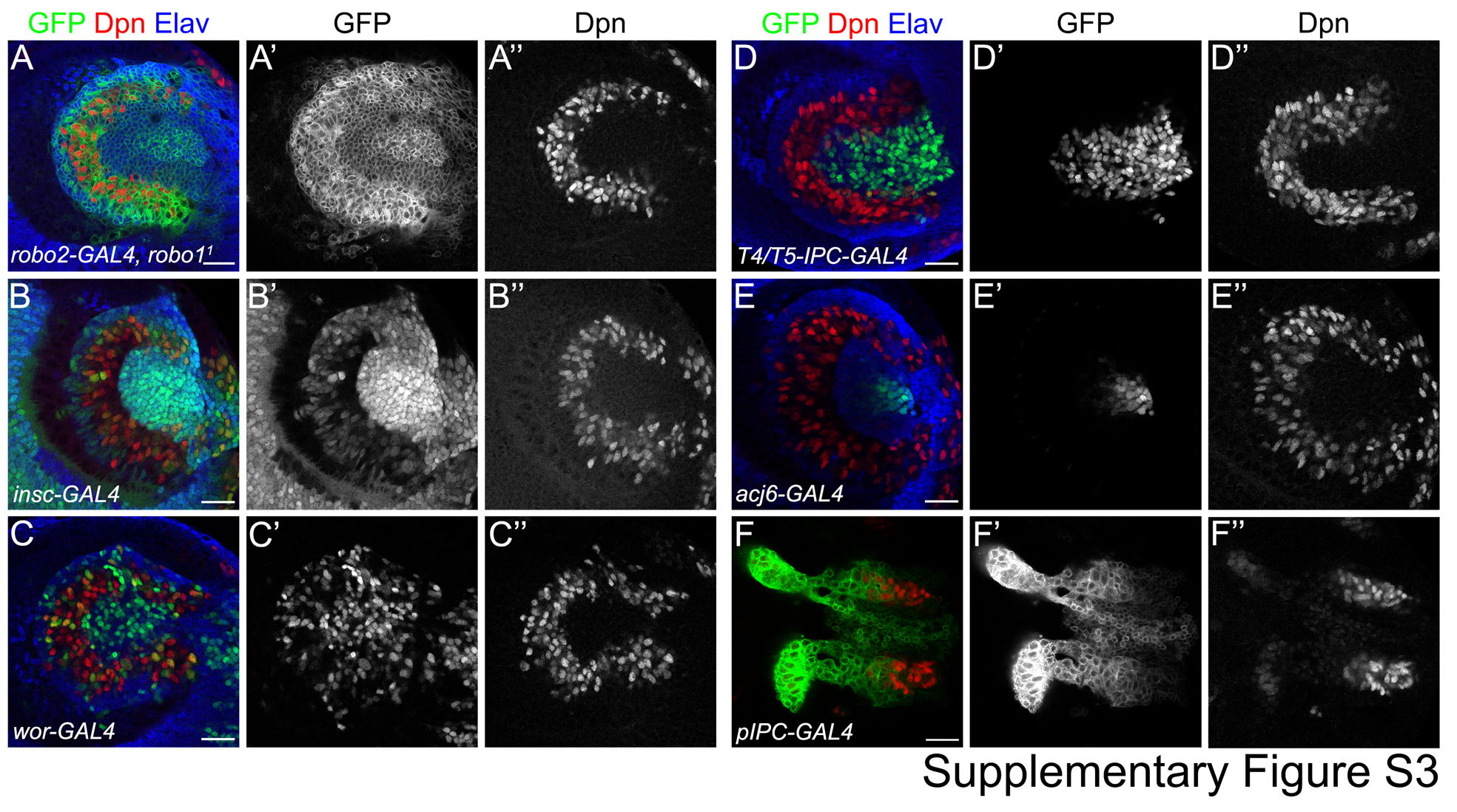

Supplement: Supplementary Figure 3 — Expression of GAL4 drivers in the larval IPC. (A–F″) Larval optic lobes stained for GFP (green, gray), Dpn (red, gray) and Elav (blue), using different GAL4 drivers to target all the cell populations in the IPC. (A–A″) robo2-GAL4, (B–B″) insc-GAL4, (C–C″) wor-GAL4, (D–D″) T4/T5-IPC-GAL4, (E–E″) acj6-GAL4, and (F–F″) pIPC-GAL4. (A–E″) Lateral and (F–F″) frontal sections. Scale bars represent 20 μm. [file Image_3.TIF]

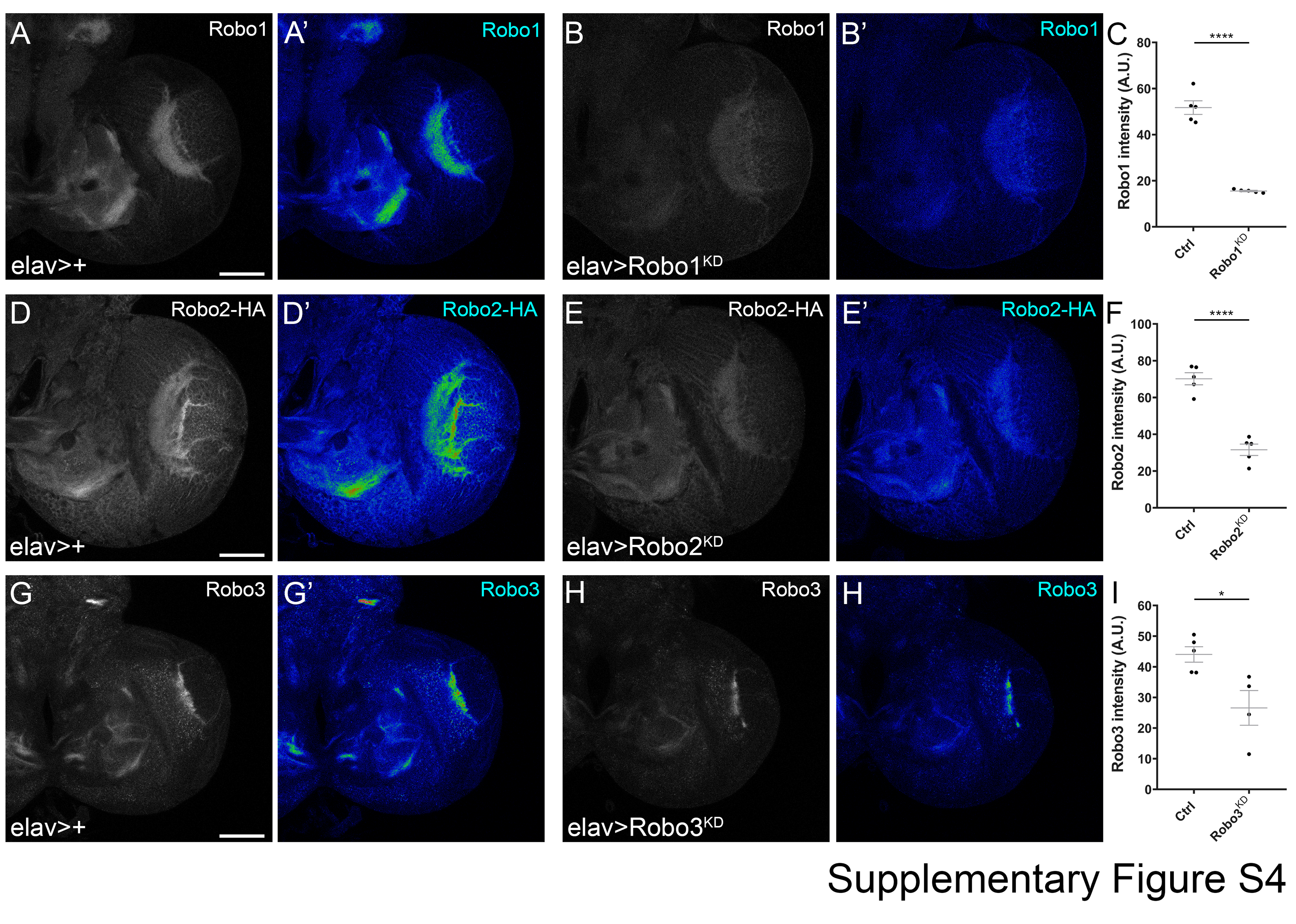

Supplement: Supplementary Figure 4 — Robo knockdown efficiency. Larval optic lobes stained for (A–B″) Robo1, (D–E′) Robo2-HA and (G–H′) Robo3. elav-GAL4 animals were crossed to (A,A′,D,D′,G,G′) w1118 (control), (B,B′) UAS-shRobo1 (Robo1KD), (E,E′) UAS-shRobo2 (Robo2KD) and (G,G′) UAS-shRobo3 (Robo3KD). (A′,B′,D′,E′,G′,H′) are pseudocolored images of Robo intensity. Scale bars represent 50 μm. (C,F,I) Graphs showing intensity quantifications of Robo1, Robo2 and Robo3 signal in control and knockdown conditions. Student’s t-test was performed. P-value are (C) 3.59 × 10^−4^, (F) 3.26 × 10^−4^, (I) 0.0189. [file Image_4.tif]

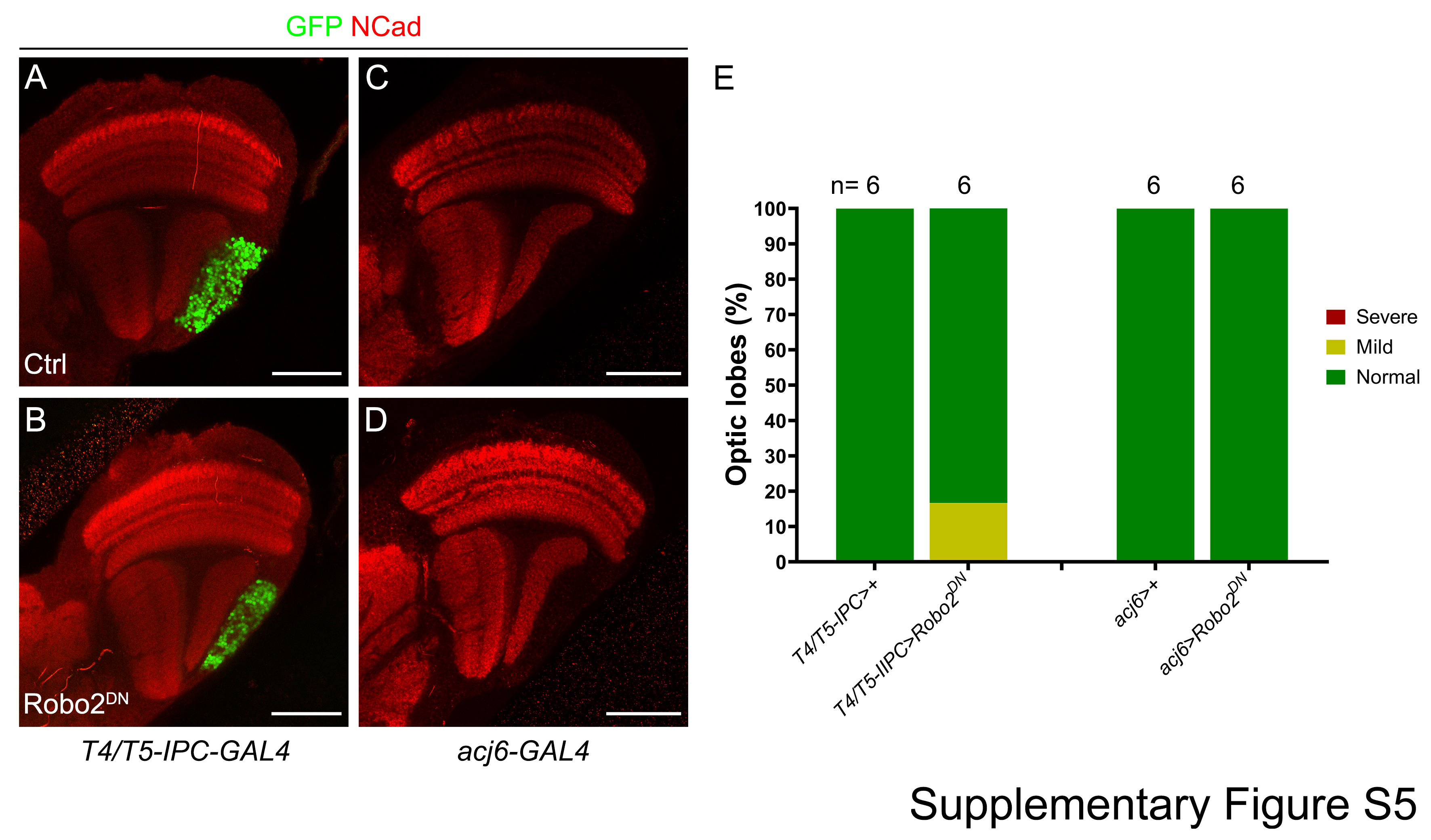

Supplement: Supplementary Figure 5 — Loss of Robo2 in neurons does not affect the morphology of the lobula plate. (A–D) Horizontal sections of adult optic lobes stained against GFP (green) and NCad (red). (A,B) T4/T5-IPC-GAL4 and (C,D) acj6-GAL4 crossed to panels (A,C) w1118 and (B,D) UAS-robo2ΔC (Robo2DN). (E) Quantification of the frequency of morphological defects in the lobula plate. Scale bars represent 50 μm. [file Image_5.TIF]

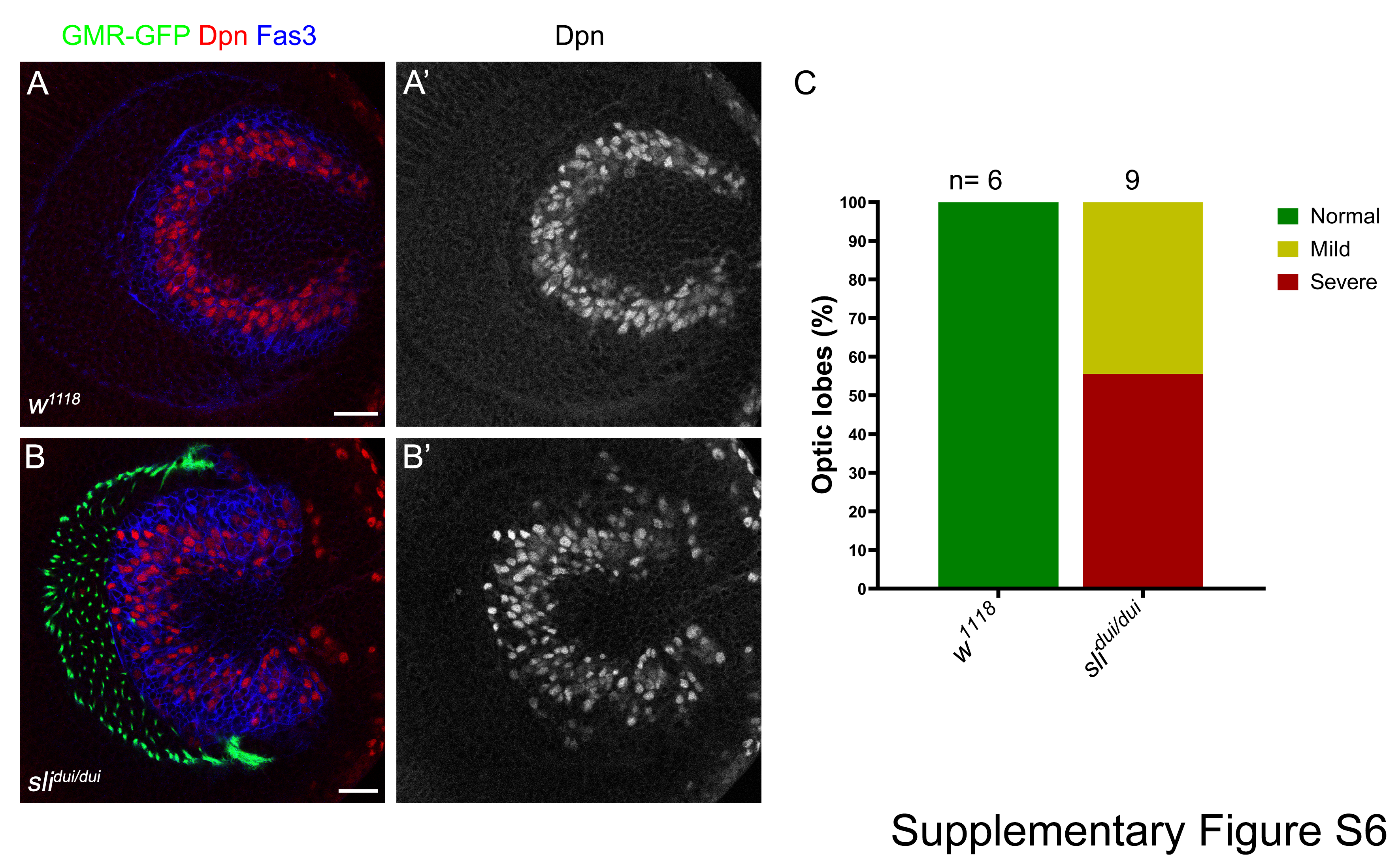

Supplement: Supplementary Figure 6 — d-IPC architecture is affected in sli mutant animals. (A–B′) Distal sections of larval optic lobes of panels (A,A′) w1118 and (B,B′) slidui/dui, GMR-GFP animals. Brains were stained for GFP (green), Dpn (red, gray) and Fas3 (blue). (C) Graph showing the frequency of disruption in the organization of the d-IPC neuroblasts. Scale bars represent 20 μm. [file Image_6.TIF]

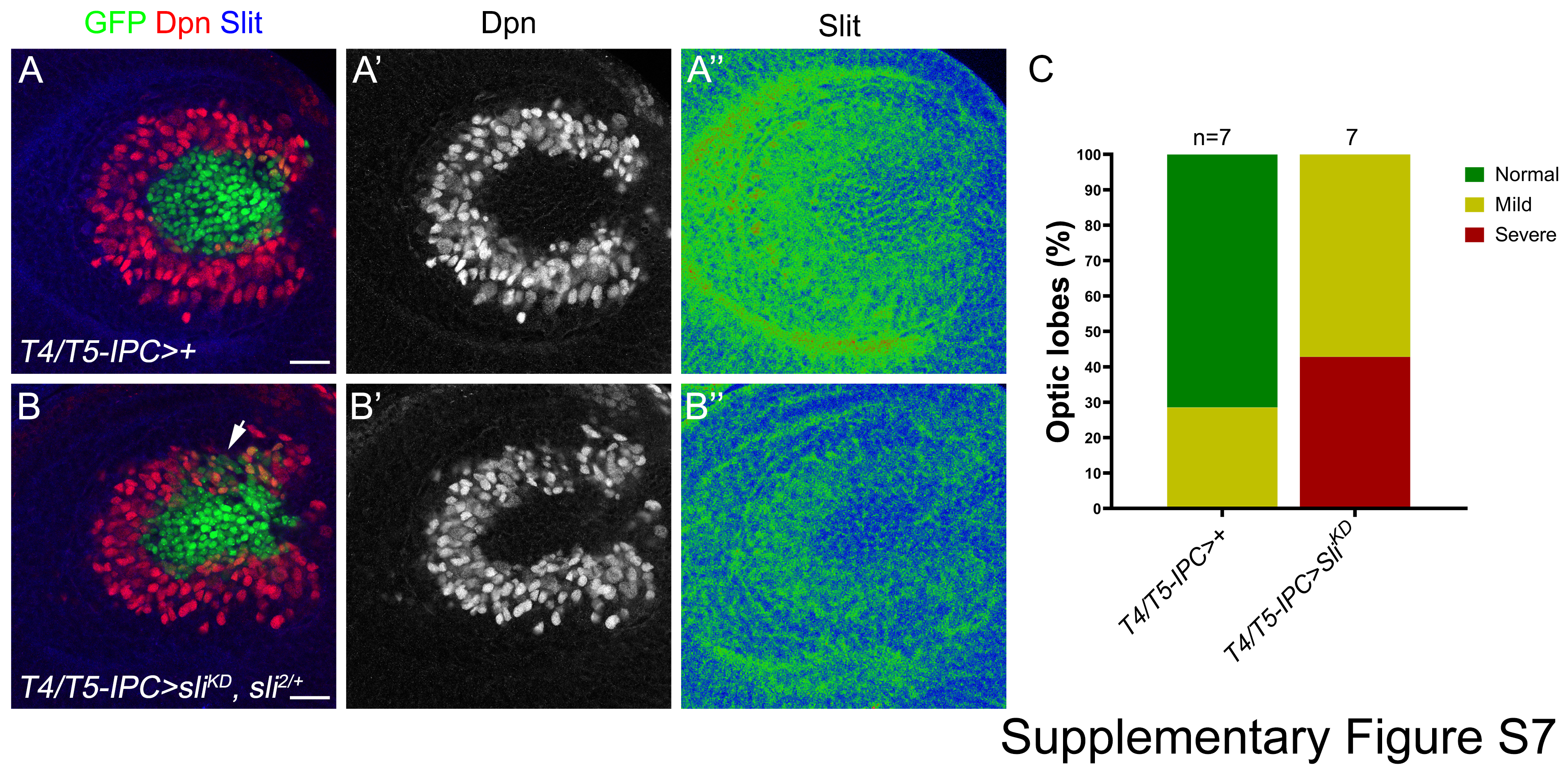

Supplement: Supplementary Figure 7 — Slit is required in T4/T5 neurons for neuroblast organization. (A–B″) Lateral sections of larval optic lobes stained against GFP (green), Dpn (red, gray) and Slit (blue, pseudocolored). T4/T5-IPC-GAL4, UAS-Dicer2, UAS-Stinger were crossed to panels (A,A″) w1118 and (B,B″) sli2/+, UAS-Sli-RNAi. Arrow shows GFP-positive neurons invading the neuroblasts horseshoe region. (C) Graph showing the frequency of brains containing neurons mislocalized in the neuroblast region. Scale bars represent 20 μm. [file Image_7.TIF]

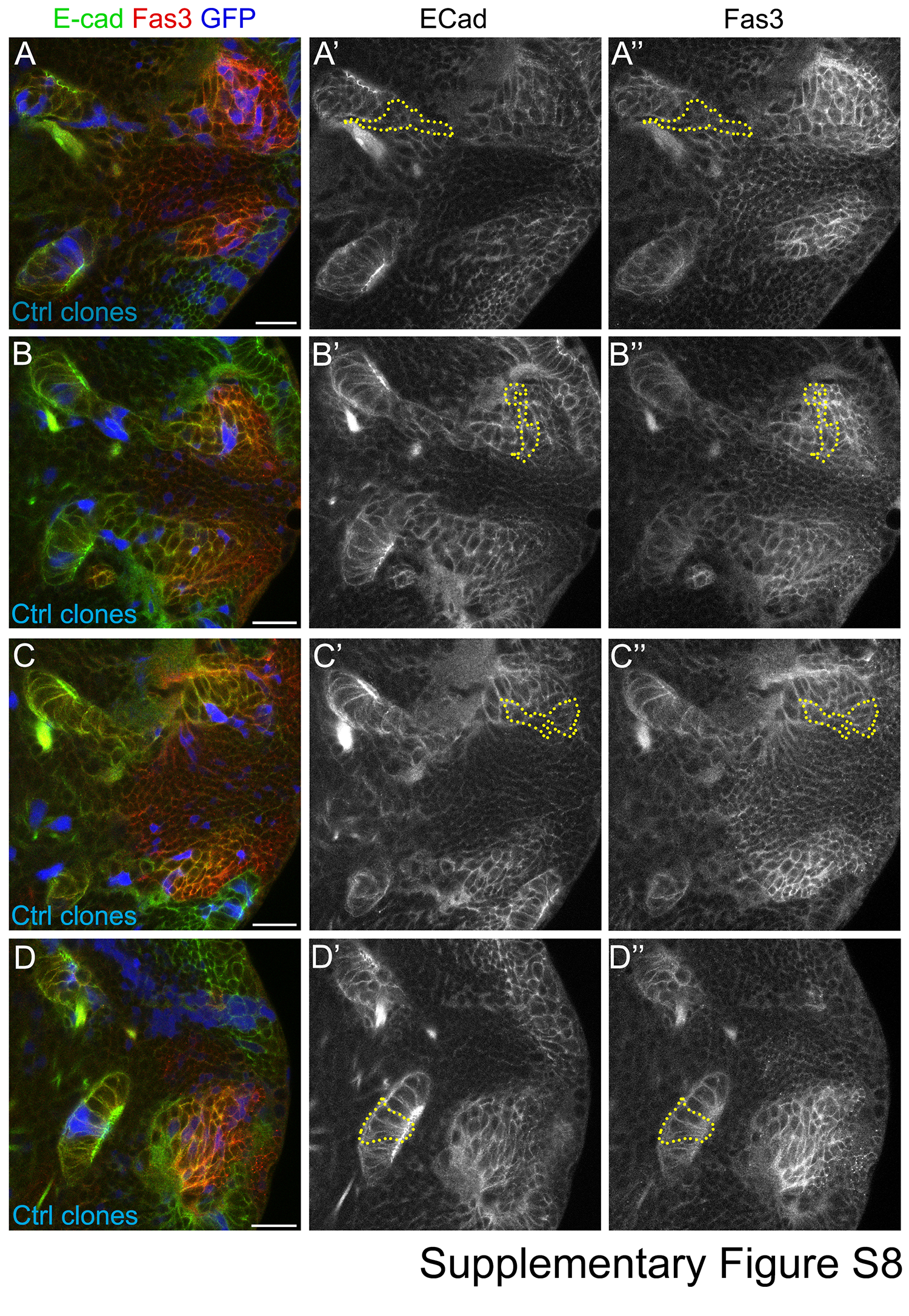

Supplement: Supplementary Figure 8 — Wild type clones. (A–D″) Larval optic lobes with control clones marked by UAS-GFP (blue), were stained for ECad (green, gray) and Fas3 (red, gray). Clones at different stages of the IPC differentiation are shown. (A–A″) p-IPC neuroepithelial/migratory progenitor clone p-IPC neuroepithelial clone, (B–B″) neuroblast clone, (C–C″) neuroblast/neuronal clone, (D–D″) p-IPC neuroepithelial. No changes in Fas3 or ECad levels were observed. Scale bars represent 20 μm. [file Image_8.TIF]

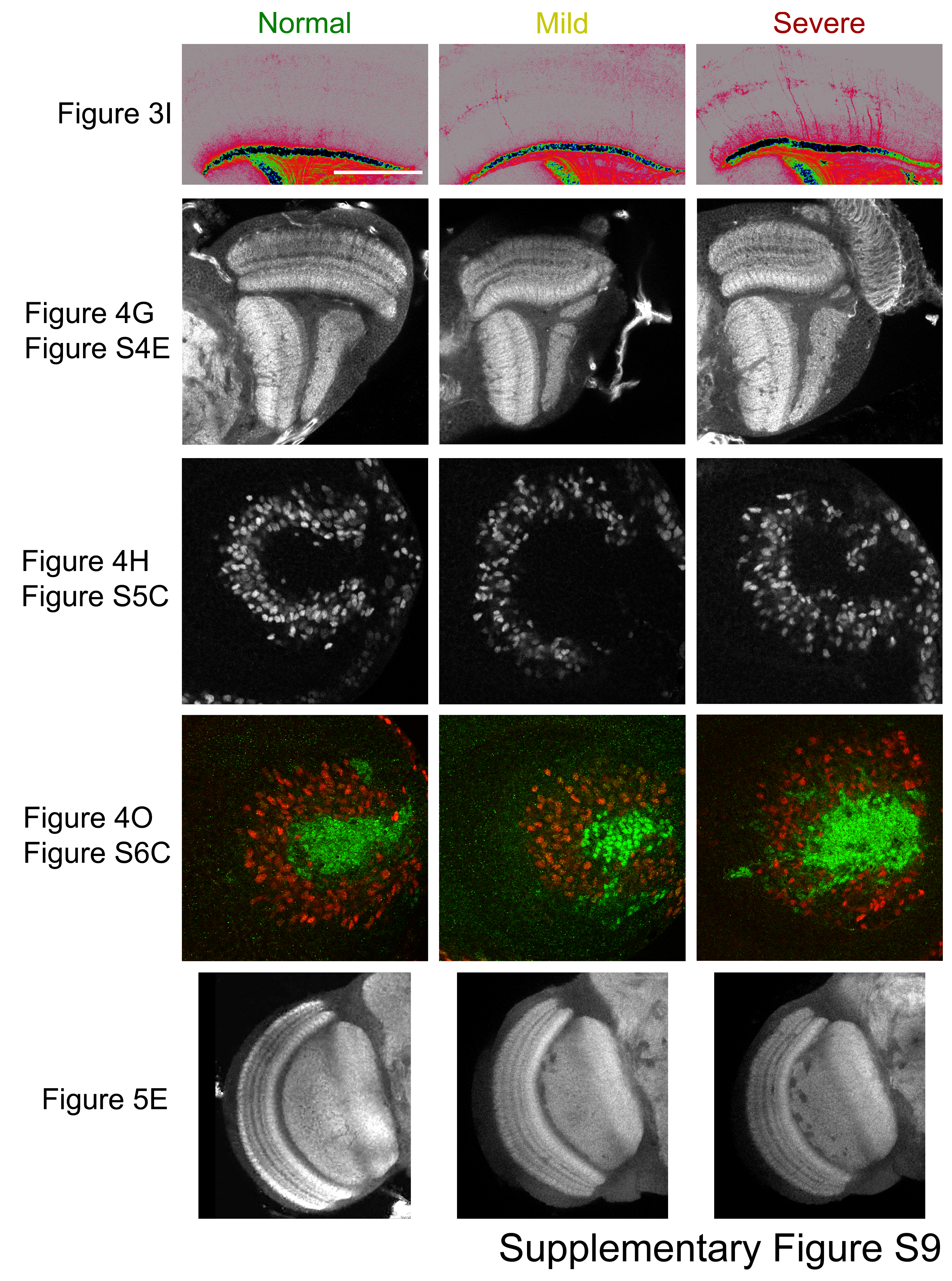

Supplement: Supplementary Figure 9 — Examples of phenotypes. Reference images used to determine the penetrance of the phenotypes in different experiments. [file Image_9.TIF]
